# Long term expression of bicistronic vector driven by the FGF-1 IRES in mouse muscle

**DOI:** 10.1186/1472-6750-7-74

**Published:** 2007-10-28

**Authors:** Camille Allera-Moreau, Aurélie Delluc-Clavières, Caroline Castano, Loïc Van den Berghe, Muriel Golzio, Marc Moreau, Justin Teissié, Jean-François Arnal, Anne-Catherine Prats

**Affiliations:** 1 Institut national de la santé et de la recherche médicale (INSERM), U858, Toulouse, France; 2 Université Toulouse III Paul Sabatier, institut de médecine moléculaire de Rangueil, équipe n°11, IFR31, Toulouse, France; 3 Laboratoire de biophysique cellulaire, institut de pharmacologie et de biologie structurale UMR 5089, CNRS, Toulouse, France; 4 Centre de biologie du développement, UMR 5547 CNRS, Toulouse, France; 5 Centre hospitalier universitaire de Rangueil, Toulouse, France

## Abstract

**Background:**

Electrotransfer of plasmid DNA into skeletal muscle is a promising strategy for the delivery of therapeutic molecules targeting various muscular diseases, cancer and lower-limb ischemia. Internal Ribosome Entry Sites (IRESs) allow co-expression of proteins of interest from a single transcriptional unit. IRESs are RNA elements that have been found in viral RNAs as well as a variety of cellular mRNAs with long 5' untranslated regions. While the encephalomyocarditis virus (EMCV) IRES is often used in expression vectors, we have shown that the FGF-1 IRES is equally active to drive short term transgene expression in mouse muscle. To compare the ability of the FGF-1 IRES to drive long term expression against the EMCV and FGF-2 IRESs, we performed analyses of expression kinetics using bicistronic vectors that express the bioluminescent *renilla *and firefly luciferase reporter genes. Long term expression of bicistronic vectors was also compared to that of monocistronic vectors. Bioluminescence was quantified *ex vivo *using a luminometer and *in vivo *using a CCD camera that monitors luminescence within live animals.

**Results:**

Our data demonstrate that the efficiency of the FGF-1 IRES is comparable to that of the EMCV IRES for long term expression of bicistronic transgenes in mouse muscle, whereas the FGF-2 IRES has a very poor activity. Interestingly, we show that despite the global decrease of vector expression over time, the ratio of firefly to *renilla *luciferase remains stable with bicistronic vectors containing the FGF-1 or FGF-2 IRES and is slightly affected with the EMCV IRES, whereas it is clearly unstable for mixed monocistronic vectors. In addition, long term expression more drastically decreases with monocistronic vectors, and is different for single or mixed vector injection.

**Conclusion:**

These data validate the use of bicistronic vectors rather than mixed monocistronic vectors for long term expression, and support the use of the FGF-1 IRES. The use of a cellular IRES over one of viral origin is of particular interest in the goal of eliminating viral sequences from transgenic vectors. In addition, the FGF-1 IRES, compared to the EMCV IRES, has a more stable activity, is shorter in length and more flexible in terms of downstream cloning of second cistrons. Finally, the FGF-1 IRES is very attractive to develop multicistronic expression cassettes for gene transfer in mouse muscle.

## Background

Gene delivery to skeletal muscle is a promising strategy for the treatment of muscle disorders such as myopathies [1,2]. Furthermore, molecules containing secretion sequences can be expressed in muscle tissue and targeted to pathologies residing in other organs. Muscle is a highly vascularized tissue, often regarded as serving endocrine functions. By expressing therapeutic concentrations of secreted proteins, such as angiogenic and neurotrophic factors, in the muscle, a number of therapeutic applications can be envisioned. Skeletal muscle has been used for the production of IL-10 in gene therapy against atherosclerosis, coagulation factors against haemophilia, erythropoietin against anaemia and influenza protein for vaccination [3-6]. Electrotransfer of alpha-2 laminin into dystrophic mouse muscle successfully produced laminin around the sarcolemmal membrane without extended muscle damage [7]. Recently, gene therapy against Duchenne muscular dystrophy was shown to be enhanced by the combined delivery of IGF-1 along with microdystrophin [8]. Such combinatorial delivery strategies will depend upon the development of optimized cassettes designed to co-express multiple transgenes.

While electrotransfer of plasmid DNA produces less protein as compared with viral vectors, there remain several advantages such as reduced toxicity, increased safety, and easier/cheaper production methods [9,10]. Electrotransfer of DNA into mouse muscle for gene therapy applications benefits from additional advantages such as tissue accessibility, efficiency of DNA uptake and long term transgene expression [11,12].

Internal Ribosome Entry Sites (IRESs) enable vectors to produce multiple products from a single transcriptional unit, eliminating the loss of gene expression due to promoter competition or counter-selection [13]. IRESs, RNA elements that permit cap-independent translation, were first discovered in viral RNAs but have also been found in several cellular mRNAs mostly encoding control proteins such as transcription factors, growth factors or proteins involved in apoptosis [14-16]. Previous work in our laboratory has shown that cellular IRESs are highly regulated *in vivo *and possess tissue specific activities [17-19]. IRESs are of particular interest for gene therapy since they could be used to co-express multiple proteins from a single mRNA [13,20-22].

Non invasive real-time analysis of molecular events in intact living animals is crucial for many applications such as gene therapy studies [23-25]. Bioluminescence quantification and imaging exploit the emission of visible photons generated by energy-dependent luciferase catalysed reactions. Firefly luciferase (LucF) converts luciferin to oxylucyferin, through an ATP-dependent hydrolysis, which emits photons detected and quantitated with low-light, charge-coupled device (CCD) cameras. Such cameras can be cooled to -80°C or -120°C to reduce thermal noise and increase their sensitivity [26]. Applications developed with these technologies are capable of non-invasively monitoring luciferase expression in living mouse muscle [11,27].

Due to alternative transcription initiations, the FGF-1 mRNAs possess 4 different 5'UTRs, three of which contain IRESs [28]. Compared to other cellular IRESs, the FGF-1 IRES A (referred to as FGF-1 IRES in the text) has a strong activity in mouse muscle. To investigate the efficiency and the stability of this IRES as a tool to drive long term transgene expression in mouse muscle, we used the "Lucky Luke" bicistronic vector, previously validated in cell culture [28]. A Lucky Luke bicistronic vector containing the FGF-1 IRES was compared to bicistronic vectors containing either the EMCV or FGF-2 IRES for expression time courses extending up to 30 days. Expression of the bicistronic vector bearing the FGF-1 IRES was also compared to that of monocistronic vectors coding for LucR or LucF, injected alone or co-injected. Plasmids were electrotransferred into *tibialis anterior *mouse muscle and quantification of bioluminescence was performed by *ex vivo *measurement with a luminometer as well as non-invasive *in vivo *imaging with a CCD camera.

Our data demonstrate that the FGF-1 IRES is as efficient as the EMCV IRES, with a more stable activity, for long term expression of bicistronic plasmids in mouse muscle, while the FGF-2 IRES is very weak. Surprisingly, expression of monocistronic plasmids expressing LucR of LucF is different when they are injected alone or together, and in both cases the decrease of expression is more drastic than with the bicistronic plasmid bearing the FGF-1 IRES. This validates the use of bicistronic vectors to optimize the control of long term gene co-expression. The FGF-1 IRES is thus a very attractive molecular tool to co-express molecules of interest for gene therapy. In addition, we demonstrate that *in vivo *imaging with a CCD camera is quantitative and a more reproducible measurement of transgene expression than *ex vivo *measurement, which requires animal sacrifice during the experiment.

## Results

### Time course of luciferase expression from IRES-containing bicistronic vectors after electrotransfer into mouse tibialis anterior muscle

**Figure 1:**
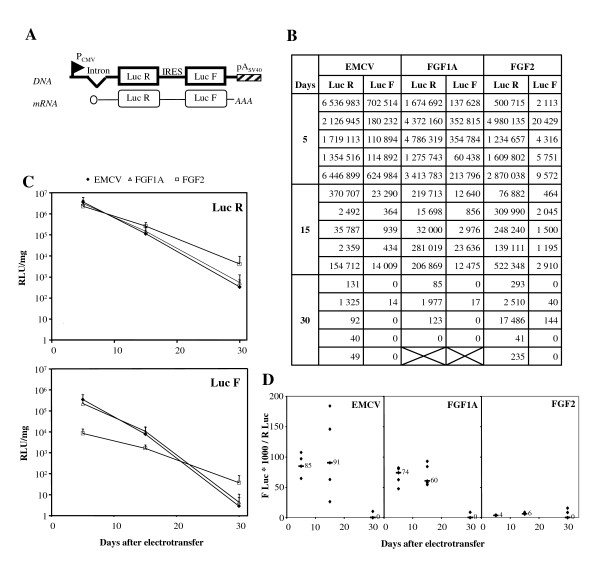
**Quantification of LucR and LucF activities in tibialis anterior muscle from C57BL/6J mice after bicistronic vector electrotransfer. Comparison between IRESs from EMCV, FGF-1A and FGF-2**. **A. Schematics of bicistronic expression vectors used to produce LucR and LucF/F+**. The bicistronic cassette, transcriptionally regulated by the cytomegalovirus (CMV) promoter, encodes LucR in the first cistron (cap-dependent translation) and LucF (Fig. 1, 2) or LucF+ (Fig. 3, 4, 5) in the second cistron (IRES-dependent translation) [28, 36]. **B. Bioluminescent signal quantification**. *Tibialis anterior *muscle was taken from mice 5, 15 or 30 days after electrotransfer. LucR and LucF activities were measured from muscle extracts using a luminometer. LucR and LucF activities were expressed in Relative Luminescent Units per milligram of grinded muscle (RLU/mg) (see Mat. & Meth). Each value corresponds to an individual mouse from an experimental group (n = 5). **C. Mean of bioluminescent signal quantification**. Mean values of the LucR (top) and LucF (bottom) activities have been represented for each experimental group presented in Fig. 1B (RLU per mg of muscle ± sem, n = 6). **D. IRES activity**. It was obtained from the values shown in fig. 1B, using the following formula: LucF × 1000/LucR. For each time point, the median value was noted with a "-". No IRES activity could be calculated in the absence of detectable LucR activity.

The "Lucky Luke" bicistronic vectors used in this study contain IRESs of different origins (EMCV, FGF-1 or FGF-2) located between the LucR and LucF cistrons (Fig. 1A). The bicistronic mRNAs express LucR in a cap-dependent manner and LucF by an IRES-dependent mechanism. Thus, both LucR and LucF are expressed from a single mRNA and IRES activity can be estimated by measuring the ratio of LucF to LucR.

"Lucky Luke" vectors were electrotransferred into the *tibialis anterior *muscle of C57BL/6J mice. At the specified time points following electrotransfer, groups of five mice (one group for each IRES) were sacrificed and their *tibialis anterio*r muscles were removed. LucR and LucF activities were quantified using a luminometer and normalised per milligram of muscle (Fig. 1B and 1C).

All three vectors showed an overall decline in LucR expression over time, suggesting that the vector expression is not stable (Fig. 1C). LucR activities had the same order of magnitude at day 5, between 0.5 and 6.5 × 10^6 ^RLU/mg of muscle depending upon individual mouse variations (Fig. 1B). This consistency persisted through day 15, however we observed a significant difference at day 30, where LucR expression was one order of magnitude superior for the FGF-2 vector (Fig. 1C). While the experiment was intended to be conducted over a period of 60 days (data not shown), very few animals produced significant LucR activity after day 30.

The IRES regulated expression of the second cistron, encoding LucF, followed the same slope as the LucR first cistron, for the three vectors (Fig. 1C). IRES activities (as measured by the ratio of LucF/LucR), remained stable until day 15 (Fig. 1D). EMCV IRES activity is very heterogenous compared to the activities obtained with the FGF-1 or FGF-2 IRESs. While the EMCV and FGF-1 IRESs showed activities within the same order of magnitude, the FGF-2 IRES activity was 10 to 20 fold lower. The kinetics could not be followed after day 30 as the expression level of LucF became undetectable.

These data show that bicistronic vectors allow expression of two transgenes with a stable ratio, depending on the IRES activity which does not change in long term versus short term vector expression. The FGF-2 IRES activity was very low *in vivo*, compared to that of EMCV or FGF-1 IRES. The FGF-2 IRES containing vector, which expressed less LucF, appeared to be more stably expressed with respect to the vectors containing EMCV or FGF-1 IRES.

### IRES activities are independent of transgene expression levels

**Figure 2:**
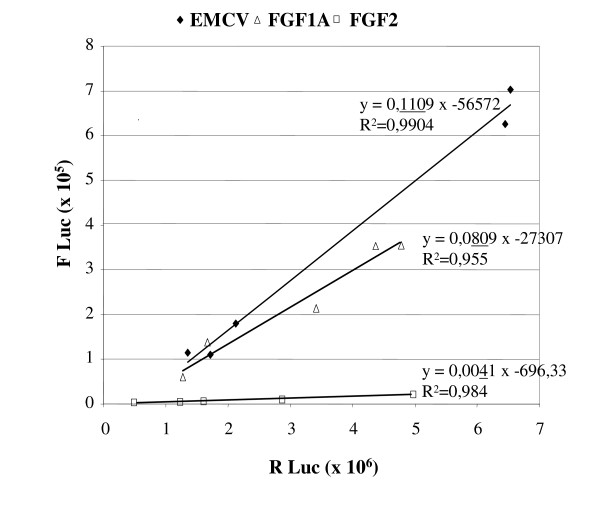
**LucF versus LucR quantification in tibialis anterior muscles of C57BL/6J mice at day 5 after electrotransfer**. For each IRES, LucF activity is expressed *versus *LucR activity to assess the correlation between the two activities. Linear regressions are shown with the regression coefficient R^2^. The slopes of the regression straight lines are proportional to IRES activities at day 5.

Linear regression analysis is another way to estimate the putative effect of transgene expression levels on IRES activity and LucF/LucR ratio. Since gene transfer efficiency is different for each mouse examined (LuR activity reflecting transgene expression), the correlation between LucF and LucR activities at day 5 were evaluated for each mouse individually. A plot of LucF activity versus LucR activity is shown in Fig. 2. At day 5, linear regression analysis revealed an excellent correlation between LucF and LucR activities when measured in the same mouse (R^2 ^= 0.9904 for EMCV, R^2 ^= 0.984 for FGF-2, R^2 ^= 0.955 for FGF-1). These results underline the fact that LucF and LucR are produced by the same mRNA, with a constant ratio. The straight line slopes are proportional to the IRES activities (0.111 for EMCV, 0.081 for FGF-1 and 0.004 for FGF-2 IRES, respectively).

### Expression of LucF+ in mice tibialis anterior muscles detected by CCD camera imaging

An important problem encountered in the previous experiments is the impossibility to follow IRES activity in a given individual mouse. This incited us to develop *in vivo *imaging methods to follow luciferase expression on live animals. Quantification of luciferase expression *in vivo *by bioluminescence imaging was performed using a CCD camera, which measures photon emission in live animals. The vectors described in Figure 1 were assayed for LucF activity using the CCD camera, but as this technique is less sensitive than the luminometer, LucF activity was difficult to detect (data not shown). Thus we replaced LucF by LucF+ (a stabilised form of LucF) in all bicistronic vectors, resulting in an approximate 10-fold increase in LucF activity (data not shown).

**Figure 3:**
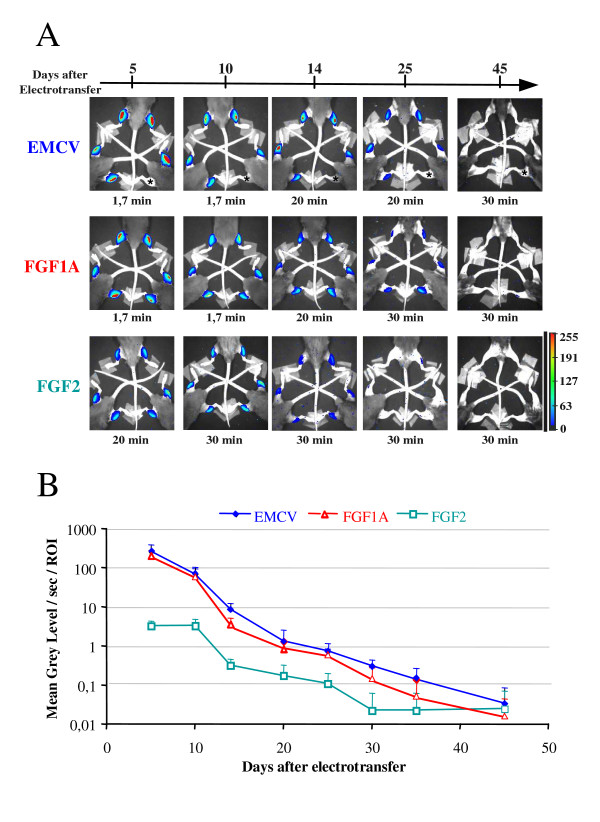
**Visualisation of LucF+ in mice after bicistronic vector electrotransfer in tibialis anterior muscle of C57BL/6J mice and intraperitoneal injection of D-luciferin**. **A. Imaging of bioluminescent signal detected with a CCD camera**. LucF+ activity was measured in mice electrotransferred with bicistronic luciferase vectors (expression of LucF+ is under the translational control of EMCV, FGF-2 or FGF-1A IRES). Images are shown in pseudocolors. Time of exposure for each image and a pseudocolor scale are represented. *: non electrotransferred muscle. **B. Mean of bioluminescent signal quantification**. Signal quantification was expressed in mean grey level/sec/ROI* (± sem). For each time point, n = 8 for EMCV, n = 10 for FGF-2 and n = 8 for FGF-1A. * Region Of Interest, corresponds to the bioluminescent area of the electrotransferred muscle.

Bioluminescent images displayed as a pseudocolor image overlaid on a grey scale reference image of the mouse muscle revealed an intense signal arising from the muscle (Fig. 3A). A non-electrotransferred muscle is indicated with a star (EMCV panel). A scale of pseudocolor is shown on the lower right side of the image panel. The integration times required to quantify LucF+ signals varied from 1.7 to 30 minutes (Fig. 3B). In correlation with the data of Fig. 1, the bioluminescence intensity from the muscles decreased as a function of time. EMCV and FGF-1 muscles revealed a high bioluminescent mean signal at day 5 (265 and 207 mean grey level/s/ROI respectively), which followed similarly decreasing kinetics until day 45 (0.03 and 0.01 mean grey level/s/ROI respectively). As in Fig. 1, muscles expressing the FGF-2 IRES containing vector showed a weaker signal at the beginning (3,32 mean grey level/s/ROI respectively) but, after decrease, showed a stabilisation between day 30 and day 45 (0,02 mean grey level/s/ROI).

### FGF-1 and FGF-2 IRESs provide a more stable LucF+/LucR ratio than EMCV IRES in long term expression

**Figure 4:**
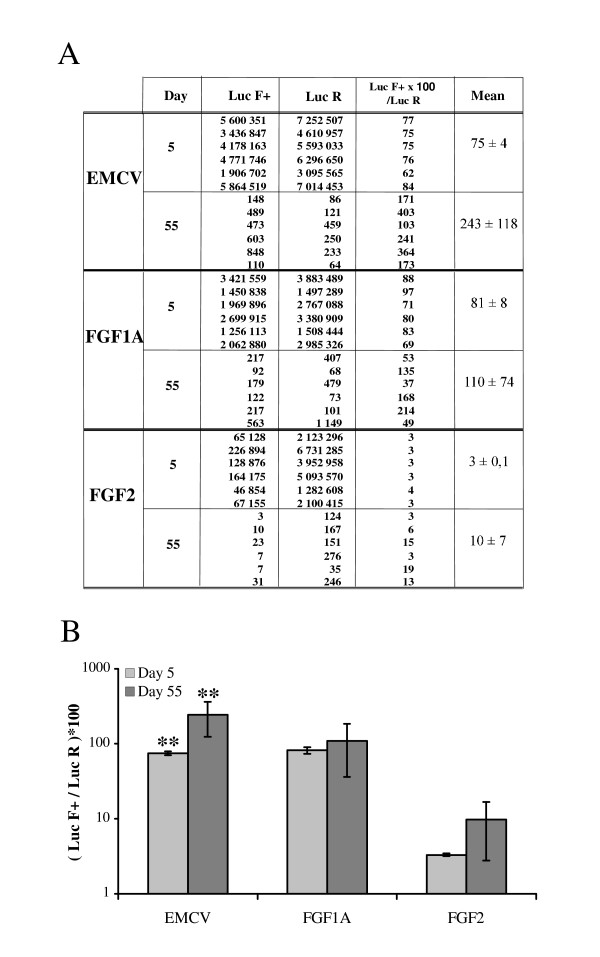
**Quantification of LucR and LucF+ activities in mouse muscle 5 and 55 days after electrotransfer**. LucR and LucF+ activities were measured at days 5 and 55 with a luminometer. **A. Bioluminescent signal quantification**. LucR and LucF+ are expressed in RLU/μg total protein. Each value corresponds to one muscle. **B. Mean of IRES activities**. It was determined as the ratio of LucF+/LucR multiplied by 100 (± sem, n = 6). Statistical anaysis, p = 0.0022 for EMCV, p = 1 for FGF1A and p = 0,0931 for FGF2.

To check the correlation between the data obtained with CCD imaging and that obtained with the luminometer, analysis of LucR and LucF+ activities were performed *ex vivo *using the luminometer at day 5 and at day 55 (Fig. 4).

At day 55, both LucR and LucF+ activities were about 10^4 ^times inferior to that observed at day 5 (Fig. 4A). This decrease correlated well with the data obtained at day 45 with the CCD camera. The LucF+/LucR ratios were not significantly different for the FGF-1 and FGF-2 IRESs at day 5 or 55, indicating that the activities of these cellular IRESs remain constant during long term expression (Fig. 4B). In contrast, the EMCV IRES activity significantly increased at day 55.

Altogether these data show that the use of FGF-1 and FGF-2 IRESs in expression vectors allows to maintain the ratio of two gene products independently of the vector expression level and of the time after gene transfer. In contrast, the EMCV IRES activity is less stable. This study validates the FGF-1 IRES as an efficient IRES that can be used for gene transfer in muscle.

### Long term expression of transgenes is improved with bicistronic compared to monocistronic vectors

**Figure 5:**
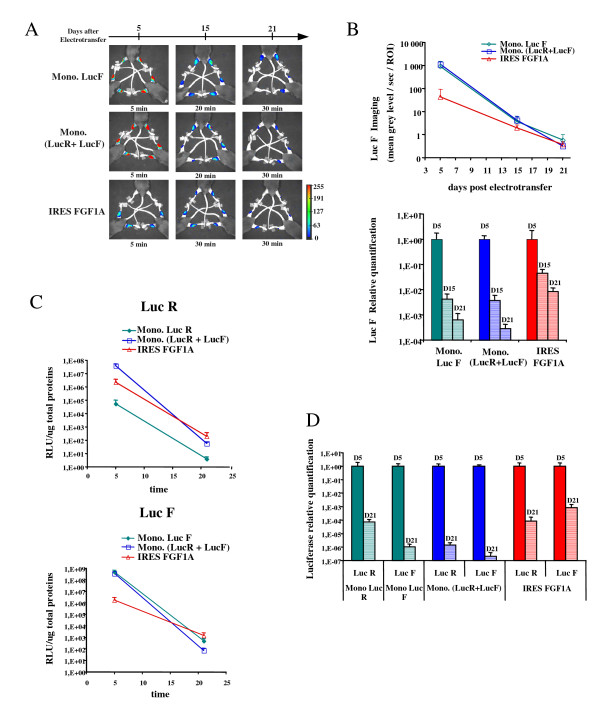
**Comparison of LucR and LucF+ monocistronic versus bicistronic vector expression after electrotransfer in mouse muscle**. Mice were electrotransferred with either 30 μg of monocistronic plasmid encoding LucR or LucF+ (Mono LucR or Mono LucF, respectively), or with a mixture (30 μg + 30 μg) of the two plasmids (Mono LucR + LucF), or with 30 μg of the bicistronic plasmid containing the FGF-1 IRES (IRES FGF1A). LucR and LucF+ activities were measured from 5 to 21 days after electrotransfer. **A. Detection of the LucF+ signal with the CCD camera**. LucF+ activity was detected at days 5, 15 and 21 in live animals as in Figure 3. Images are shown in pseudocolors. Time of exposure and a pseudocolor scale are represented. **B. LucF+ signal quantification**. On the top panel, the values detected with the CCD camera for each time point are expressed in mean grey level/second/ROI (mean ± sem, n = 6). The bottom panel shows the LucF+ relative quantification at day 5 (D5), day 15 (D15) and day 21 (D21) for each vector. **C. Luciferase activities quantification at day 5 and 21 after electrotransfer**. Two groups of mice were sacrificed at day 5 and 21, and muscles lysates were used for luciferase activity quantification using the luminometer. LucR (top panel) and LucF+ (bottom panel) are expressed in RLU/μg total proteins (mean ± sem, n = 6). **D. Bioluminescent signal relative quantification**. Luciferase relative quantification was obtained for each vector at day 5 (D5) and day 21 (D21).

It has often been claimed that monocistronic vectors are expressed with a higher efficiency than bicistronic vectors. Indeed, a previous report has shown efficient transgene co-expression seven days after plasmid co-injection [29]. In order to determine if there is an advantage in using bicistronic vectors rather than mixed monocistronic vectors for long term expression, plasmids encoding either LucR or LucF were electrotransferred separately or mixed, and their expression was compared to that of the bicistronic vector bearing the FGF-1 IRES. The time course was followed up to 21 days (Fig. 5). Luciferase activities were either monitored by CCD camera imaging (Fig. 5A and 5B) or quantified with the luminometer (Fig. 5C and 5D). Both analyses showed that the bicistronic vector expression was lower than that of monocistronic vectors at day 5, except for the monocistronic expressing LucR alone (Fig. 5A, 5B, 5C). However this difference disappeared at day 15 and was inverted at day 21: luminometer quantifications indicated that, for both LucR and LucF, the bicistronic vector was more efficiently expressed, at day 21, than the monocistronic vectors injected separately or together. In addition, the relative decrease presented in Fig. 5B and 5D histograms shows that monocistronic vector expression decreased more quickly when co-injected than injected alone.

As regards the LucF to LucR ratio, it was not significantly modified with the bicistronic vector, whereas LucF expression decreased more drastically than LucR with the monocistronic vectors. In addition, LucR expression level was radically weaker for the monocistronic vector single injection than for co-injection.

These data clearly show that the monocistronic vectors, although providing a higher expression at day 5, are less efficient than the bicistronic vector for a long term expression. In addition, the difference observed for the monocistronic vector injected alone or co-injected renders its expression unpredictable, in contrast to the bicistronic vector containing the FGF-1 IRES that provides a stable transgene ratio in long term expression.

## Discussion

In this study, we have analyzed the *in vivo *expression of bicistronic and monocistronic vectors following naked DNA electrotransfer in *tibialis anterior *mouse muscle. In particular, we have compared the activities of two cellular IRESs, FGF-1 and FGF-2 IRESs, to that of the widely used EMCV IRES. Our data indicate that although vector expression drastically decreases after 55 days, FGF-1 and FGF-2 IRESs maintain a constant ratio of the levels of expression of the two genes encoded by the vector independently of the expression level and of the time after gene transfer. In contrast, the EMCV IRES activity significantly increases during the time course. Strikingly, monocistronic vectors, although more efficiently expressed in short term, showed a long term expression lower than that of the bicistronic vector containing the FGF-1 IRES. Furthermore, expression of the monocistronic plasmids was different when injected alone or together and the transgene ratio unstable. These data demonstrate the advantages of IRES-containing bicistronic vectors, and particularly of the recently identified FGF-1 IRES, as biotechnological tools for long term co-expression of transgenes in skeletal muscle. This study also shows that the CCD camera is a powerful tool to follow vector expression and IRES-dependent translation in live animals.

Vector expression is maximal, for monocistronic as well as bicistronic plasmids, 5 days after electrotransfer. Luciferase activities are however rapidly decreasing. This is surprising as previous reports demonstrate a persistence of LucF expression until 19 months [12,30]. Bloquet et al also describe a long-lasting expression of luciferase, which reached a peak value at 7 days after electrotransfer and a plateau value that persisted for at least 270 days [12,31]. However, the strong decrease of luciferase expression observed in our study corresponds to the observation of Durieux et al who have demonstrated that the duration of plasmid expression is highly dependent on the promoter origin. These authors observe a decrease of luciferase activity of 96% at day 21 compared to day 5 with the CMV promoter, whereas the decrease is only 19% with the cytochrome c promoter [32]. In our study, we show that the monocistronic vector expression more drastically decreases than that of bicistronic vectors. At day 5 the bicistronic vector is about ten times less expressed than the co-injected monocistronic vectors, whereas at day 21 the bicistronic vector is significantly more efficiently expressed than the two monocistronic ones. A possible mechanism to explain this difference may be that the stronger expression of the monocistronic vectors may favor their elimination by the immune system. We can thus hypothesize, consistent with the Durieux et al study, that the duration of plasmid expression could be related to its level of expression. Consequently the strategy of using plasmid vector with a moderate expression seems interesting for long term transgene expression. Multicistronic vectors thus offer the possibility to express moderate amounts of several molecules of interest that can have synergistic effects, rather than huge amounts of a single molecule which can generate unwished effects.

An important point discovered by the present study is that IRESs can provide a constant ratio of transgene expression. At day 5, our linear regression analyses revealed excellent correlations between LucR and LucF for all three IRESs, demonstrating that the *in vivo *IRES activity is not influenced by the level of transgene expression, which varies between the individual mice (Fig 2). FGF-1 and FGF-2 IRES activities are not significantly modified up to 55 days after DNA electrotransfer, whereas EMCV IRES activity slightly increases with time. In contrast, co-injection of two monocistronic vectors shows drastic variations of the transgene ratio. This can probably be explained by a possible competition between the two vectors that could lead to quicker elimination of one of them if the encoded protein is more antigenic or has some toxical effect. A previous report has shown that co-injection of two monocistronic vectors allows a high degree of co-expression in muscle after seven days [29]. Here we demonstrate that bicistronic vectors with IRESs, especially FGF-1 and FGF-2 IRESs, provide an advantage over co-injected monocistronic vectors for long term co-expression of transgenes.

The CCD camera appears as a precious tool to follow the kinetics of LucF activity, but proved problematic with respect to LucR monitoring, which was hindered due to substrate precipitation and CCD camera sensitivity (not shown). This camera allowed us to detect and quantify muscle bioluminescence from the same mouse over a time course. The use of the CCD camera reduces the number of sacrificed animals per experiment, sometimes by as much as one order of magnitude, because it allows the comprehensive assessment of each animal over the entire duration of the process under investigation [33]. Furthermore, the LucF+ activities observed with the three IRESs exactly correlate with those observed with the luminometer, indicating that the camera quantitatively reflects LucF+ expression (not shown). However, sensitivity is inferior with the camera compared to the luminometer, as previously reported (Fig. 5 and 6) [34].

Finally, one may find it unclear how would this translate into a meaningful gene therapy, as detection of luciferase is very sensitive and the signal amplified. Of course, detection based on enzymatic activity does not mean that the amount of protein produced would be biologically meaningful in all cases. However, expression of luciferase in bicistronic vectors is very useful in preclinical assays for gene therapy as it allows to monitor the expression of the gene of interest on live animals. Furthermore, in various gene therapy applications such as cancer or lower limb ischemia, gene therapy approaches may use secreted molecules (angiogenic or anti-angiogenic factors) that are active in very low amounts. In such cases it is not necessary to flood the organism with therapeutic molecules but more interesting to obtain long term expression of moderate amounts of several therapeutic molecules having synergistic effects. Bi- or multicistronic vectors are thus particularly attractive to co-express combinations of such therapeutic molecules.

## Conclusion

In conclusion, our data show that bioluminescence imaging *in vivo *offers a powerful tool for the quantitative real time analysis of gene expression [27,35]. We validate the advantage of using IRES-containing bicistronic vectors, rather than monocistronic vectors, for long term expression of transgenes and show that one can deduce the expression of the first cistron by monitoring the expression of an IRES-driven second reporter cistron *in vivo*. This can be applied to monitor the expression of a gene of interest.

Our results reveal the FGF-1 cellular IRES as an interesting new tool to be used in multicistronic vectors for three reasons. It is of cellular origin, an important aspect with respect to the goal of removing viral sequences from transgenic vectors. It is shorter and more flexible, thus easier to use for cloning genes of interest than the EMCV IRES. Finally it is equally efficient as the EMCV IRES in terms of expression kinetics in muscle and its activity is more stable when driving transgene long term expression. Together the data presented here will enable the construction of new expression cassettes that express combinations of molecules for therapeutic purposes.

## Methods

### Recombinant vectors

All bicistronic vectors contain the expression cassette "*Renilla *Luciferase (LucR) – IRES – Firefly Luciferase (LucF)" under the control of the cytomegalovirus (CMV) promoter with the SV40 polyA signal. The IRES sequence is of viral (EMCV) or cellular origin (FGF-2 or FGF-1). A chimeric intron of human β-globin origin is present between the CMV promoter and the expression cassette [36]. LucR was subcloned from pRL-CMV, LucF from pGL2 and LucF+ from pGL3 (Promega, France). The plasmids used for the study of EMCV, FGF-1 or FGF-2 IRES were pCREL, pCRF1AL and pCRFL respectively [17,28]. The monocistronic plasmids CMV-LucR and CMV-LucF+ had the same vector skeleton as the bicistronic vectors. The plasmids used for injection in mice were purified with a MAXI EndoFree plasmid purification kit (QIAGEN). For *ex vivo *bioluminescence quantification, bicistronic vectors contained LucF. For *in vivo *bioluminescent imaging, LucF was replaced by LucF+ because of its higher stability.

### Mice

6-week old female C57BL/6J@Rj were purchased from Janvier laboratories. They were housed in the animal facility of the IFR31 (Toulouse, France) and cared for in conformity with the guidelines of the INSERM Ethics Committee. Mice were anesthetized by intraperitoneal injection of Ketamine (125 mg/kg Body Weight) and Xylazine (10 mg/kg Body Weight) solutions and mice legs were depilated using a depilation cream (Veet).

### In vivo tibialis anterior muscle electrotransfer

Groups of 5 mice received an intramuscular injection with naked DNA in saline solution followed by electrotransfer. 30 to 45 μg of bicistronic vector (in a 30 μl final volume) was injected intramuscularly using an Insulin syringe (300 U, Myjector u-100 insulin, Terumo). A conducting gel was applied on both sides of the injected muscle (Eko gel, Asept. InMed S.A.). The electric pulses were delivered using a generator (ECM 830, BTX) and Tweezerhode electrodes (522, 10 mm diameter, BTX) placed on both sides of the *tibialis anterior *muscle. Eight 20 ms pulses were applied with a voltage setting of 200 V/cm at a frequency of 2 Hz. One muscle, which did not receive any DNA injection, was included as a control.

### Quantification of LucF and LucR expression in tibialis anterior muscle lysate with a luminometer

At time points between 5 and 60 days after electrotransfer, mice were anesthetized and *tibialis anterior *muscles were removed and stored at -80°C. Lysates were generated by grinding the tissue in Passive Lysis Buffer (Promega, France) using an Ultra-Turrax T25 (Janke and Kunkel, IKA^R ^Labortechnik). Quantification of *ex vivo *bioluminescence was performed with a luminometer (Centro LB960, Berthold) using the Dual-Luciferase^® ^Reporter Assay (Promega, France) according to the manufacturer's instructions.

Raw data were obtained from the MicroWin 2000 software. The mean control value (corresponding to non electrotransferred muscle) was multiplied by 2 and subtracted from each sample value. Results were then expressed either in Relative Luminescent Unit (RLU)/mg of muscle weight or in RLU/μg of total protein.

### Quantification of total protein with Dc Protein Assay

The amount of total protein in the ground muscle was quantitated using the Dc Protein Assay (Bio-Rad). Briefly, 20 μl of S reagent was mixed with 1 mL of A reagent to obtain the A' reagent. 25 μl of this A' mix was added to 1 μl of the sample in a 96 well plate (Nunclon TM) and 200 μl of B reagent was added. After 10 minutes incubation time, the OD was read at 690 nm. In parallel, a BSA range standard was used to correlate the OD with the total protein amount in the sample.

### In vivo bioluminescence imaging and quantification

*In vivo *bioluminescence imaging was conducted using a cooled CCD camera (cooled to -80°C) mounted on a dark box chamber with a camera controller, a camera cooling system and a computer system for data acquisition and analysis (deep cooling BTW 512 S/N, camera 1394 ORCA II, Hamamatsu Photonics). 5 days after electrotransfer (and up to 30 days or 55 days), mice were anesthetized and mice legs were depilated. D-luciferin was then injected intraperitoneously (50 mg/kg body weight, Luciferin-EF™, Promega France) and after 5 min incubation time mice were placed in the dark box chamber. During image acquisition animal body temperature was regulated using a heating plate integrated within the dark box chamber. A grey scale body surface image was collected in the chamber under dim illumination, followed by acquisition and overlap of the pseudocolor image representing the spatial distribution of detected photon counts emerging from active luciferase within the animal. An integration time of 1.6 to 30 min was used for luminescent image acquisition. The grey scale images and bioluminescence color images were superimposed using the Simple PCI software (Hamamatsu Photonics). Signal intensity was quantified as the mean of grey level per second of time exposure within a region of interest (ROI) prescribed over the muscle using the Simple PCI software. Levels of luminescence are indicated by pseudocolors on a scale of 0 to 255 units. The control ROI chosen on the abdomen permitted measurement of the background, which is subtracted from the sample values.

### Statistical analysis

Statistical analysis was performed using the program Prism^® ^(GraphPad Software Inc.). Mann Whitney tests (two-tailed) were achieved to compare IRES activities at day 5 and day 55 after gene transfer.

## List of abbreviations used

FGF-1: fibroblast growth factor 1

FGF-2: fibroblast growth factor 2

EMCV: encephalomyocarditis virus

IRES: internal ribosome entry site

LucR: *renilla *luciferase

LucF: firefly luciferase

ROI: region of interest

CCD: charge-coupled device

CMV: cytomegalovirus

OD: optical density

DNA: deoxyribonucleotidic acid

RNA: ribonucleotidic acid

## Authors' contributions

CAM and ADC had an equal contribution in the gene transfer *in vivo *and bioluminescence experiments, as well as in figures achievement and manuscript draft.

CC and LVDB participated in the bioluminescence experiments.

MG carried out a part of the DNA electrotransfer experiments.

MM helped to optimize the *in vivo *bioluminescent detection technique.

JFA and JT participated in the study design.

ACP was the main designer and coordinator of the study, and achieved the redaction of the manuscript.

All authors read and approved the final manuscript.
